# Anthropological perspectives on CKDnt in Mexico: time for a paradigm shift on the social determinants of health

**DOI:** 10.3389/fneph.2023.1155687

**Published:** 2023-06-12

**Authors:** Ciara Kierans, Cesar Padilla-Altamira

**Affiliations:** ^1^ Institute of Population Health, University of Liverpool, Liverpool, United Kingdom; ^2^ Unidad Occidente, Center for Research and Higher Studies in Social Anthropology, Guadalajara, Mexico

**Keywords:** anthropology, CKDnT, Mexico, paradigm shift, social determinants of health

## Abstract

In Mexico, the kidneys of individuals in poor and marginalized communities are failing with little warning and no explanation. Commonly referred to as chronic kidney disease of non-traditional origin (CKDnt), this new variant of kidney disease cannot be accounted for by conventional or discrete etiological explanations, but is instead understood to be a consequence of economic development, environmental degradation and precarious working and living conditions. Drawing on two interconnected ethnographic studies, and the intertwining problems of causation and care, this paper will (1) document the social conditions of disease emergence around Lake Chapala, Central Mexico, and (2) follow the haphazard routes kidney patients take to access resource-intensive biotechnical treatments. Its aim is to both challenge and reconceptualize social determinants *as* social relations in order to fully account for the profoundly contextual, temporal, and dynamic character of this condition, and to rethink opportunities for care and intervention.

## Introduction

The paper starts with an ethnographic example focused on Agua Caliente. Agua Caliente, situated on the shores of Lake Chapala, west central Mexico, is one of a cluster of small towns and villages in the municipality of Poncitlán. The municipality is home to a population of approximately 53,000 people who endure disproportionately higher rates of chronic kidney disease (CKD) than elsewhere in the wider state of Jalisco (1). In Agua Caliente, residents’ kidneys fail without explanation, and this affects both the young and old. The Martinez family have been particularly afflicted, that is, the two teenage sons, David and Eduardo, mother Lola, her brother and his daughter, Lola’s niece, have all been diagnosed with an unexplained variant referred to as chronic kidney disease of unknown origin (CKDnt). CKDnt is different from CKD, primarily because it cannot be attributed to established causes, such as diabetes or hypertension. Instead, it is impacted by climate change and environmental degradation (2). When speaking about their condition, the Martinezes talk about having *riñones chiquitos* (small kidneys), a term that either means that they were born with small kidneys—a congenital condition, known as renal hypoplasia and which can lead to CKD—*or* that their kidney disease was very advanced prior to diagnosis, resulting in a shrinkage of the organ, thus making it difficult to perform a biopsy to establish a cause. Talking about *riñones chiquitos* is idiomatic of the uncertain status of kidney disease among those in lakeside communities.

Life for the Martinez family is precarious. Across the three generations who make up their household, livelihoods are supported by a mix of fishing, construction, agricultural work, and domestic cleaning, supplemented by subsistence farming. Their small house is an “*autoconstrucción*” (one built incrementally *via* a series of extensions, as necessary and budget allowing). It is overcrowded, which makes caring for a loved one with kidney disease, on dialysis, or recovering from transplant surgery profoundly challenging.

Those who live in Agua Caliente are a mix of mestizo and Indigenous peoples. They are poor. They depend on their local environment for their dietary staples of corn and beans, fish from the lake, and whatever can be grown or domesticated in their household gardens. This environment is fragile. Lake Chapala is heavily contaminated, yet central to everyday life. It provides a place to bathe, wash clothes and household items, catch fish, and draw water. In the absence of a local refuse service, it also serves as a site for waste disposal. Food is cooked on homemade wood-burning stoves, which operate throughout the day in poorly ventilated spaces, creating the conditions for poor pulmonary health, a comorbidity that sits alongside cancers, diabetes, chronic diarrhea, violence, alcoholism, and occupational harms, all of which compete with kidney disease for medical care and attention (1, 3). These conditions are made worse by difficulties accessing doctors, dentists, pharmacies, midwives, and other health professionals. For the young people who have been identified as especially vulnerable to developing CKDnt, their need for appropriate and timely healthcare is structurally unattainable. Why? Because those who live in Agua Caliente are uninsured and have few social entitlements to healthcare. To understand the implications that this has for inequalities and kidney disease, we must grasp the wider sociopolitical relations within which healthcare is organized, both as a feature of contemporary Mexico and as an outcome of its history and the socio-environmental conditions within which CKDnt emerges.

In Mexico, the conditions under which CKDnt emerges are complex. It is associated with intense work in strong heat, for example in the agri-industries or among construction workers, as well as in environmentally degraded settings where there is a risk of exposure to heavy metals such as mercury, arsenic, cadmium and uranium and widespread use of pesticides (4, 5). High rates of CKDnt in Mexico have been reported across the states of Aguascalientes, San Luis Potosi, Jalisco, Veracruz, and Tlaxcala (1, 4, 6), with the disease disproportionately affecting poor and uninsured Mexicans.

## Aim

This paper addresses CKDnt as a disease of poverty, inequality, and precarity through a lens of multiple and overlapping limits: the limits of causal models, the limits of health care, the limits of ecosystems, and the limits of our human imaginations to consider health and intervention “otherwise“ (7)—to rethink the limitations of current public health approaches and open up the potential for transformative possibilities. We argue that transformative possibilities can only emerge from collaborative standpoints. As one small step in this direction, we reflect on opportunities for medical anthropology and public health: two domains with shared commitments to public health, social justice, and structural inequalities, but with critically different methodological positions on how the category “the social” determines or shapes health outcomes, positions that are often in contention rather than collaboration. Writing from the perspective of an anthropologist, the aim of this paper is twofold: (1) to offer a brief characterization of the ecosocial conditions of CKDnt emergence in the Lake Chapala region attending to the interlocking concerns of causation and care with a view to identifying opportunities for collaboration and intervention, and (2) to unpick the category of the “social” and ask how an anthropological sensitivity to context and temporality can temper the social determinism of public health.

## Methodological approaches

The ethnographic research that this paper is based on reflects two interrelated projects. The first began in 2011, following and documenting uninsured kidney patients in the city of Guadalajara as they attempted to access resource-intensive biotechnical care for CKD. In the absence of recognized pathways to renal care, the project was organized around (a) the practices and experiences of uninsured kidney patients and their families as they constructed their own routes to care, and (b) the work of doctors, nurses, pharmacies, medical suppliers, governmental bodies, charities, and voluntary associations, among other public and private interests—all of which are centrally involved in supporting and/or providing care. A total of 134 participants participated in the project (8). The study analyzed the relations between the state, the market, and the healthcare system made visible by ethnographic work (3). As the project came to a close, concerns about an unexplained CKD variant emerged. In fact, many of those interviewed had no medical explanation as to why their kidneys were failing, and a significant proportion of them came from communities around Lake Chapala, some 70 kilometers from Guadalajara. A second pilot project was developed in 2017 to follow the cases of 12 families from the public hospital they were treated in back to the communities they lived and worked in. These 12 cases were entry points that soon progressed to include others with a stake in the condition: other family members, friends, and neighbors; health professionals; environmental activists; and politicians, among others. With them we mapped CKDnt emergence across a 20-year period from 2000 to 2020, drawing on local narratives, media, and scientific reports (9). With a further pilot study, and a small team of researchers (anthropologists, historians, medical, and public health specialists), we took social histories of health and environmental changes across three generations. In the absence of formal epistemic accounts of CKDnt, our immediate priority was to follow the condition by staying close to the “actors” themselves (10), documenting the condition as a vernacular concern, and attending to their experiences and everyday environmental encounters. Our ethnographic work made use of what the historian Carlo Ginzburg (11) calls trace data: vernacular descriptions, media reports, incomplete hospital records, environmental activist accounts, and varied and variable scientific reports, and linking them together in evidentiary chains. It was also modulated by what was already known about CKDnt in other parts of the world, in addition to our interdisciplinary sensitivities to health–environmental relations as slow-moving effects (12). The studies were supported by ethics approvals at Hospital Civil de Guadalajara, Fray Antonio Alcalde (Mexico) and the University of Liverpool (UK). Pseudonyms are consistently used in publications. The research adhered to the ethical codes of practice set out in the requirements of the American Anthropological Association and the Association of Social Anthropologists, in the UK, which reflect the particularities of conducting ethnographic fieldwork.

Our longer-term aims are to integrate the diverse knowledges (vernacular and scientific) that have grown around CKDnt to comprehensively examine the social and environmental conditions of its emergence. We want to find new ways of thinking about etiology that shift emphasis from the discrete and linear relations of cause and effect or “social determinants” to encompass the rich intersections between human practice, environmental change, and the human body—and in doing so, to rethink opportunities for intervention and care.

### CKDnt: on the limits of care and causation

What follows is not a traditional report on study findings but a set of reflections and characterizations of key ecosocial conditions and relations of CKDnt emergence as a means of rethinking the social determinants of health. A central assumption guiding this paper is that “causation” and “care” are deeply entangled, contingent elements. How we understand causation (broadly speaking) shapes our strategies for intervention and care (13). How we might ultimately imagine care reflects our responses to the “imperfect work” of science and time itself (14). As one nephrologist put it when talking about the urgency of CKDnt: “this is not a disease we can dialyze or transplant our way out of - we need to find other ways”. In other words, we need to imagine care “otherwise”. In what follows, we focus attention on the cluster of impoverished communities disproportionately affected by CKDnt in Poncitlán on the northern shores of Lake Chapala. We elaborate the intertwining problems of disease causation and care in two parts: part 1 includes discussion on the political economies of healthcare, the costs of care, and the sacrificial labors of care. Part 2 focuses on socio-environmental etiologies. At the heart of both is a concern with power and the social relations of health. It is to these concerns that we now turn.

### The political economies of healthcare: state–market–health relations

In Mexico, as in many other countries around the world, the conditions and relations of labor determine access to social insurance and set the terms for and levels of social protection, welfare, and healthcare. Access to and organization of renal services – as with other health services—is fragmented, characterized by profound inequalities, and administered by way of a complex quasi-insurance-based social security system linked to labor market position (15). Under this system, and at the time of study, private-sector salaried workers and their families—approximately 44 percent—were covered by the Instituto Mexicano de Seguro Social (IMSS, the Mexican Institute of Social Security). Civil servants and federal workers approximately another 5% of participants—were covered by the Instituto de Seguridad y Servicios Sociales de los Trabajadores de Estado (ISSSTE, the Institute of Social Security and Services for Civil Servants). Smaller social insurance systems exist for those working in nationalized industries such as PEMEX (Mexican Petroleum). Private health insurers cater for highly paid workers, who constitute about 2% of the population (16). These insurance systems are all linked to different networks of hospitals and clinics. For those not covered by any formal insurance—approximately half of the population—they were reliant on services provided at a subsidized cost by the clinics and hospitals of the Secretaría de Salud (the Ministry of Health). These are run with no guaranteed suite of services and at significant cost. Services are thus limited and heavily reliant on out-of-pocket payments made at the point of use. A proportion of this constituency did have access to Seguro Popular, a form of ‘popular health insurance’, although this has since been discontinued and replaced by the Instituto de Salud para el Bienestar (INSABI). Seguro Popular, unlike IMSS, ISSSTE, and other social insurance systems, provided no comprehensive support for forms of dialysis or transplantation. Descriptions of Mexico’s fragmented healthcare, are, to some extent, rhetorical, because in a flexible labor market, citizens move through various forms of social protection and insurance as work is lost or gained, or indeed abandoned in the face of systemic bottlenecks, long waits, or dissatisfaction with services. Each healthcare system provides its own idiosyncratic route through primary, secondary, and tertiary services. Under these conditions, social security is insecurely anchored, temporary, and revisable (3, 17).

Scholarship on the welfare state provides an important resource for understanding how the emergence, consolidation, and organization of Mexico’s hybrid corporatist/neoliberal welfare state works to frame the instabilities of health insurance in the country. (18–20). The Mexican welfare state is a perpetual work in progress. It is a governmental “*autoconstrucción*”, its elements are continuously being adapted but rarely adequately finished. In practice, it works to prevent large proportions of the country’s poor and economically insecure population from receiving social security entitlements. This is partly due to a growing reliance on new private markets in healthcare and their accompanying infrastructures, and a splicing of public and private provision, which is increasingly problematic in many countries today and was the engine of the aforementioned Seguro Popular (21). Social protection and entitlement are also a function of history. Mexican welfare has, for much of the previous century, been characterized by a system of institutionalized brokerage that ties together the relations of capital, labor, and the state. This system of brokerage has roots in the Cardenas government of the 1930s, which sought to make the country’s largest labor unions the base of party support, promoting collective bargaining agreements that included social security benefits (19). It was ostensibly an engine of internal stratification based on the systemic favoring of vested urban interests, with the Indigenous and rural poor being locked outside systems of state support and ignored in attempts to extend them. Unequal access to healthcare is one of the many challenges faced by the people of Poncitlán today. For those attempting to access renal care, the routes to it are both haphazard and catastrophically impoverishing (3).

### The costs of care

Mexican patients and their families must negotiate a complex infrastructure of public and private healthcare providers, clinics, and laboratories. Although renal care is subsidized, it still means paying for, among other things, hospitalization, surgical procedures, routine check-ups and tests, dialysis, pre-transplant protocols, biopsy needles, stitching for wounds, disinfectant, and medications. The costs are exorbitant and need to be appreciated in relation to travel costs, dietary costs, structural costs for those on home dialysis, informal care-giving costs and, of course, the loss of formal earnings for those who can no longer work in order to care for a loved one (22). In order to meet these costs, uninsured Mexican families must amass resources in whatever way they can. They do so by lobbying healthcare professionals, politicians, and local businesses; paying insurance premiums (when possible); appealing to networks of family and friends; selling land, property, and inheritances; and begging. The labor involved in doing so does not always guarantee a positive health outcome. We have witnessed time and again families become penniless in their attempts to secure an organ transplant for a relative, only to then be unable to meet the costs of immunosuppression and aftercare.

Cost—both financial and moral—characterizes the entire renal care trajectory. In Mexico, peritoneal dialysis (PD) is the preferred option of the state (3). PD uses the peritoneum in a person’s abdomen as a membrane to remove excess fluid and toxins and stabilize electrolytes. It is predominantly performed in the home setting at significant personal and financial cost, requiring family members to take specific training, pass exams, and ultimately create a “paraclinical” space in the home to deliver it (23). In order to source costly dialysis supplies, in particular dialysate, families establish informal systems for sharing supplies and medications, or will pass them on to others if a loved one dies or undergoes a transplant. What is more, Mexico’s fragmented healthcare system militates against having a comprehensive and integrated deceased organ donation program and so relies on a system of living-related organ donation, again outsourcing to wider kin. Living-relative organ donation similarly produces an intense and intimate moral economy of supply and demand. Approximately 80% of kidneys for transplant are sourced from family members in this way (24). Living-relative organ donation is only made possible by the perceived bioavailability of “the other kidney” (25), assuming that we can all live healthily on one kidney. However, in a context of concentrated environmental harms and rising cases of unexplained kidney disease, particularly among multiple household members, the bioavailability of “the other kidney” is being called into question (3).

How people manage costs relies on social relations, which manifest obligation, solidarity, and altruism. They constitute “moral economies”: Ways to cope with the limitations of healthcare for those who must negotiate their own entitlements (26, 27). Moral economies reflect the kinds of political and economic relations that provide the particular conditions for their organization and arrangement (28).

### Sacrificial economies

The labors required of families to establish renal care might not always produce healthy outcomes, but they are productive in other ways. Moral economies, such as we see in Mexico, generate significant value for others, because they pull various public and private interests into play, such as pharmaceutical companies, medical suppliers, laboratories, pharmacies, and medical staff. It is through the continual labor of Mexican patients and their families that we see not just the limits of care but what might be regarded as a sacrificial economy of care. This is required from the poorest and most insecure, particularly when forms of waged labor do not insure against the risks of sickness and when the state’s apparatus for social protections not only fails but renders their situation markedly more difficult. Such failures of social protection give rise to profound dispossession [Marx, 1970 (29) ]. They dispossess the poor of the means to sustain and reproduce life at its most fundamental and raise critical questions about what it means to establish appropriate and timely healthcare solutions for CKD when rates are not only soaring [CKD is the second leading cause of death in Mexico today (30)], but also when the conditions under which people are falling ill are increasingly uncertain and difficult to diagnose. Is it, then, possible to think about care “otherwise”? And how might an understanding of the social and environmental conditions of CKDnt emergence provide some signposts in that direction?

### Socio-environmental etiologies: understanding the social conditions of CKDnt emergence

The village of Agua Caliente takes its name from the hot springs found along the edge of Lake Chapala. Lake Chapala is Mexico’s largest freshwater lake and one of the most endangered and polluted water systems in Latin America (31). It is connected to the heavily industrialized and contaminated Lerma River, forming a watershed (Lerma-Chapala Basin) which supports approximately 20 million people (32). Industrial dumping, inadequate wastewater treatment, heavy metal contamination, in addition to the widespread use of unregulated pesticides and agrochemicals, have had direct consequences for the health of the lake and provide the context for CKDnt.

Despite the epistemic uncertainties that characterize CKDnt in formal scientific terms, those living close to Lake Chapala have little difficulty describing the condition as an outcome of a changing ecosystem and are intimately aware of the relationship between their ailing bodies and their ailing environment. Routine encounters with contaminated fish, water, the diminishing quality of agricultural produce, in addition to inadequate water management infrastructures for sewage, sanitation, irrigation, and pesticide run-off, convince locals of their etiological links with CKDnt.

When the first cases of CKDnt emerged in the early 2000s, the water level of the lake was at its second lowest in history, making visible untreated waste from industries discharged all along the Lerma Basin. Environmental scientists raised concerns about heavy metal and pesticide accumulations. Concentrations of lead, copper, and mercury were reported in the muscles and liver tissue of fish, especially carp (33, 34). Contaminated carp raised particular concerns about levels of methylmercury and its consequences for young women who may bear children. It also pitted the fishing industry, local communities, and municipal authorities against each other in efforts to preserve either livelihoods or health (3). Although carp, among other fish, is still widely eaten, concerns about the safety of doing so have only increased.

When the lake recovered in 2003, it flooded a sewage treatment plant in the nearby town of San Pedro Itzicán. The sewage treatment plant had been constructed 2 years earlier as part of a municipal plan to provide an integrated sewage network for Poncitlán. Engineers had not factored in fluctuations in lake levels and the plant was not designed to function under water. When the lake water rose, it spewed sewage into the lake and onto the shores where children played, and locals washed and shore fished. This occurred again in 2004 and 2012 as a result of heavy rainfall and has generated concerns about the links between gastrointestinal infections, diarrhea, and acute kidney injury (35), and also widespread public anger.

Water contamination is an ongoing challenge for daily consumption and food production. All modes of water access and delivery are problematic: from ground water aquifers and wells to infrastructures of piped water that regularly corrode and break. Local communities have to improvise continually. For those that can afford to do so, families purchase 20-liter plastic water *garrafones*, often produced by entrepreneurially minded locals. However, they are unregulated and like tap water, this bottled water is found to be contaminated by coliform bacteria, including *Escherichia coli*, fecal matter, and arsenic (36). Entanglements between disease, infrastructural failures, and a degrading environment are perennial sources of complaint. For over 20 years, complaints have taken the form of demonstrations, multiple community and stakeholder meetings, and a protracted human rights legal proceeding, all with little success.

In the absence of comprehensive clinical, population, or environmental evidence, vernacular knowledge remains one of few routes to understanding the complex relations linking together environmental harms and health, and repositioning etiology as an ecosocial or indeed political matter (37). Ecosocial etiologies do not of course reflect discrete causal pathways but do map out the understood and felt correspondences and juxtapositions between the rise of a condition, a local environment, and the everyday encounters of populations with that environment over time (14). In contexts of rising concerns about environmentally induced diseases, accounts grounded in local vernacular knowledges show us both where and how to look to elaborate the “social” conditions of disease emergence and potentially how to target preventative solutions accordingly. Moreover, they position local populations as collaborators (rather than recipients or victims) co-producing the kinds of evidence that might shape social, structural, and (infra)structural interventions. Our local interlocutors have taught us that rather than put health problems, such as kidney diseases, cancers, diabetes, gastrointestinal infections, and so on, into direct competition with each other, as problems with discrete causes and responses, we might focus, instead, on shared, in-common, interventions and prevention to produce farther-reaching effects. This, of course, does not exclude the quest for discrete causality—that would be a naïve dismissal of science itself—but simply challenges its privilege and recognizes that kidney diseases do not stand alone. It is, instead, the primacy of the discrete disease category that is at issue here (38). Recognizing the limits of causal narratives have direct consequences for established public health approaches and forms of reasoning. The remainder of this paper will discuss this.

### The challenge for public health: toward an interdisciplinary dialogue

CKDnt, like other environmentally induced diseases (39, 40), presents vexing challenges for public health. It challenges conventional modes of clinical reasoning and intervention and shows that we cannot draw neat lines around cause–effect relationships. CKDnt is a “wicked” problem (41): multifaceted, ambiguous, uncertain, and temporal. It is also an outcome of “superwicked” problems, such as climate change, global warming, and the complex consequences of political, economic, and industrial processes, and is one that those seeking to solve are often implicated in causing (42). A disease with no central epistemic authority, unresponsive to conventional approaches to understanding and intervention, CKDnt requires collaboration across the sciences and humanities, and with various public actors in order to produce agile and adaptive responses (43). However, doing so is far from straightforward and raises critical questions (as we can see from the above example) as to what counts as *proper* knowledge and evidence when casual explanations are limited, and what counts as appropriate care and intervention when the care for human bodies, public health, and lived environments has been compromised. These are fundamentally social questions; questions where history and context matter.

### Contesting the “social”

Anthropologists and public health scholars agree that inequalities in health are, at root, social concerns. However, these disciplines diverge in what they mean by this. In public health, the “social” finds expression in the social determinants of health (SDH), an approach which can be traced from the thinking of Frederick Engels (44) and Rudolf Virchow (45). More recently, it extends from the work of Richard Wilkinson (46, 47 and Michael Marmot (48, 49, who argued that social and historical gradients in morbidity and mortality have to be understood as outcomes of political and economic processes, a perspective that was later enshrined in WHO policy. Anthropologists and public health scholars might also agree on how the subsequent WHO Commission on the Social Determinants of Health articulates the causes of unequal health outcomes. In ‘Closing the Health Gap in a Generation’, the commission (50:1) states the causes as being:

“the unequal distribution of power, income, goods, and services, globally and nationally, the consequent unfairness in the immediate, visible circumstances of people’s lives – their access to health care, schools, and education, their conditions of work and leisure, their homes, communities, towns, or cities – and their chances of leading a flourishing life. This unequal distribution of health-damaging experiences is not in any sense a ‘natural’ phenomenon but is the result of a toxic combination of poor social policies and programs, unfair economic arrangements, and bad politics”.

As a consequence, the public health imperative lies in social transformation. However, despite the aspirational tone of the report, the SDH framework has sustained criticism: for an incapacity to translate aspiration into action; for resting too heavily on descriptive and static approaches of social processes; for producing weak analyses of power, and, as a result, for having little capacity to create sustainable interventions (51–54). Without wishing to reiterate critical appraisals on the SDH (please see 54–58), I provide a brief account of methodological and analytical differences that distinguish public health and anthropological approaches to the “social”.

Public health tends to approach the “social” as a domain in its own right, one distinct from the “technical” domain of the biological and medical sciences (59, 60), and separate from the individual, who is often the object of intervention (55). The “social” is assumed to be discrete, preestablished, unidimensional, and objective, capable of being anatomized into other discrete elements, and, as such, measurable. It is a construct that has been weakly conceptualized (51), foreclosing sociality itself, and also temporality and contextual variation (61). The “social” nestled within the SDH can be considered an artifact of the epidemiological methods and metricized modes of research used to investigate it (54, 62; 63). This is at variance to anthropological approaches that emphasize the “social” as relational, situated, and temporal. The anthropologist Emily Yates-Doerr (61: 380) points out that in assuming a linear approach to causality, the SDH addresses:

“the roots of a pre-given problem imagined to begin at a measurable point and to then advance to a predictable (i.e., determinant) place … With determinacy as an underlying metaphor, the social determinants literature is full of unidirectional arrows, producing neat but unhelpful causal relations.”

She argues that this offers prescriptive solutions that often do not result in the deep structural transformation they claim to inspire. To see the “social” as a determinant masks a non-equivalence between various markers of inequality, such as income, education, diet, and health (64). It can simplify indices of multiple deprivation that have different implications in different settings (51), thereby decontextualizing the public’s health and making it difficult to ameliorate the kinds of structural inequalities that a SDH framework is imagined to respond to, ignoring other aspects of society and social processes, for example the role of the state, governmental institutions, neoliberal policies, and corporate actors (54, 57, 63, 65, 66). As a default, public health interventions tend toward lifestyle or behavioral changes rather than social changes. This, in turn, runs the risk of stigmatizing and pathologizing those most vulnerable to poor health (64), while avoiding the thorny and tricky subject of power and the powerful.

In a heavily cited response to the CSDH’s “Closing the Gap” report, Vincente Navarro (52:15) explicitly draws attention to the apolitical nature of the report and provides a corrective to the commission’s maxim that *social inequalities kill*, revising it to emphasize, “It is not *inequalities* that kill, but those who benefit from the inequalities that kill.” He contends that inadequate attention to power is one of the core weaknesses of the SDH framework and reasons why it fails to transform the conditions of inequality. In a similar vein, Nayar and Kapoor (67:118) point out that the report smooths out social difference by giving “an impression that the ‘whole world is one family’ where responsibility for improving the social determinants of health is placed on all players whether private sector, public sector, state, government, civil societies, communities of international bodies without realizing the unequal power relations existing in the society.” This tendency to assume social and biological equivalence while flattening out power is again an artifact of method, that is, “the excessive reliance on traditional scientific ideas about generalizability” (54: 5).

If power has been backgrounded by SDH literature, it has been foregrounded in medical anthropological literatures (68–70). One example of this is the widespread use of the concept of “structural violence”. Originally conceived by John Galtung (71) and extended in scope by the physician–anthropologist Paul Farmer (70), “structural violence” reflects the coexistence of accumulations of wealth and poverty within the same political and economic systems, which create the basis for the unequal distributions of life chances and harm. That inequalities are “structured”, does not mean that they are static or universal. Structures simply reflect patterns of collective social action that have stabilized over time and in place (72), thus acquiring the appearance of permanence, regularity, and normativity. Structures are observable in economic arrangements, institutional practices, laws, policies, and so on. These patterns of collective social action also manifest materially and infrastructurally (73) and are embodied in our roads, hospitals, transportation, energy supplies, sewage, and sanitation systems.

By juxtaposing structure with violence, Farmer attends to the everyday sufferings and injustices that are embedded in the taken-for-granted patterns of the way the world is made, and which are outcomes of long histories of political, economic, and social struggle. Because they are outcomes of collective social action, structures can be changed. Structural violence encourages us to look for the relations of difference between social groups, or indeed indifference to social groups, to provide an account of inequality and social suffering. In other words, structural violence links diseases to social conditions and asks us to identify their particular features (74).

Although structural violence has proven to be an important heuristic within anthropology, Herrick and Bell (63: 304) point out that it has been criticized on precisely the opposite grounds of the SDH, “[T]hus, if the SDH framework ‘speaks of policies without touching on power’ (quoting Navarro), structural violence speaks of power without touching on policy.” In other words, structural violence can tend toward generating more “moral heat” than concerted action (75: 322). Nevertheless, it is the liveliness and social and relational force at the heart of concepts like structural violence, which we wish to propose is key to opening up the conditions within which CKDnt emerges and is a counterbalance to a social determinants perspective which tends to prefigure the “social” in advance as something discrete and measurable.

### Positioning social relations and context: a space for anthropology

Describing and understanding social relations is at the heart of anthropology. As we have seen in relation to CKDnt in the context of Lake Chapala, this means that people’s actions and practices are analyzed as part of the diverse relations in which they are enmeshed, and include relations with abstract entities such as the state, religion, and corporations and, indeed, with the material things and infrastructures that are part of their everyday environments (76). Understanding social relations, therefore, cannot be done from the outside in, by working with already formed or preestablished understandings of relations, but by following the phenomena in question and staying close to the practices and meanings of our interlocutors (77). This means attending to the manifestations, trajectories, and contexts of CKDnt and how it is encountered by those with something at stake in it (78). In that way, an understanding of the phenomenon is built up from within (79). Putting things into context and showing the implications, functional logics, and outfalls of social relations is the aim of the ethnographic exercise (80). It helps us to see the multiple modes of existence that shape our social worlds. This is political in so far as contextualizing enables an appreciation of difference rather than uniformity (81). This is a fundamental divergence between a social relations and social determinants perspective; the former privileges what is situated, local, and differentiated; the latter, what is standardized, measurable, and scalable. It is in holding up the complexities of social life, the juxtapositions, and multiple views of others that analytical insights emerge for the anthropologist (14). Said another way, to contend with the kinds of social forces that shape and condition environmentally induced diseases similar to CKDnt, we must avoid foreclosing alternatives or overdetermining our analysis with normative positions worked through in advance. We, thus, rely on approaches that embrace the liveliness of the “social”.

To structure the many different explanations which produce accounts of CKDnt, we treat the condition as a “work-site [ … ] around which different types of social actors coalesce to produce new insights, understandings and interventions” (3: 133). It is precisely because CKDnt is uncertain and unstable as a medical category that differing perspectives are critical. They also show that the development of knowledge and evidence is not neutral but is reflective of the social and political processes that go into making and stabilizing it (10). In this sense, CKDnt can be regarded as a “communicable” disease, an outcome of the forces of communication that drive its rise, spread, and understanding thereof^1^. Seeberg and Meinert (82) contend that ideas of communicability have been poorly conceptualized in health, confusing how we understand contemporary epidemics. Rethinking communicability not only requires recognition of the importance of multiple viewpoints, that is, linking vernacular and scientific knowledge together, but also of the social and political–economic forces that shape disease emergence, and disease capacity to spread in specific environments.

## Conclusions: lessons for interdisciplinary and collaborative working

Since the millennium, across the global south, there has been an upsurge in reporting the unexplained and unprecedented increase in CKD, specifically new variants of the condition, which indicate distinct changes in etiological profile (83, 84). Documenting the rise and spread of this disease is a public health imperative, as epidemiologists map its emergence and effects across Central America, sub-Saharan Africa, and parts of India, Nepal, China, Sri Lanka, and the Middle East (85). Although we have only focused attention on the intersections between environmental contamination and CKDnt, much of the research literature to date has emphasized the emergence of CKDnt in relation to climate change and work, ostensibly the relationship between heat stress and agricultural laboring (86, 87).

CKDs are thought to affect approximately 800 million people globally (88), up to 10 million of whom are estimated to die each year, a large proportion without access to medical treatment (89). Given limited and patchy population data across variable global settings, these figures are likely to be underestimates of a problem that is only likely to increase given persistent human encroachment on fragile ecosystems (87). There is much that we do not know. We do not know if CKDnt is novel, that is, a disease of our time, or indeed if it is an outcome of slow-moving ecosystemic shifts (12). We do know that Costa Rica, one of the few affected countries with reliable routine data going back to the 1970s, shows that it has been extant since then (90). We also know that CKDnt, as a disease of inequality and precarity, disproportionately affects the poor. When viewed through a SDH framework that relies on static and discrete forms of variable analysis and linear causal processes, CKDnt is difficult to contextualize. If recent experience with COVID-19 has taught us anything about environmentally induced diseases, it is that is difficult to draw neat lines around cause–effect relationships or even locate discrete diseases. We, therefore, need new paradigms of understanding, and for action that is capable of complexity and mindful of context, social relations, and temporality. Collaboration between public health and anthropology is one place to start. However, this requires paradigmatic and methodological shifts. As we can see from our example of CKDnt emergence in Lake Chapala, this means embracing contingency rather than determinacy (91). This means appreciating that health and social inequalities are situated and conditioned historically, bound up with a political economy and the work of the state. It also means appreciating the work of moral economies, that is, the kinds of social obligations and solidarities that emerge when formal welfare and social protections fail, and indeed the social encounters, vernacular knowledges, and forms of local reasoning that help us to understand the complexities of etiology and where opportunities for intervention might lie.

Thinking in terms of social relations, rather than social determinants, leads us briefly back to the work of Michael Marmot, one of the architects of the SDH. In an article focusing on historical perspectives on the SDH, Marmot reflects on lessons learned from his mentor, the social epidemiologist, Len Syme. He recalls his shock when Syme told his students that doctors have no special insight into the causes of ill health, and that giving “primacy to biology betrayed a classic misunderstanding of the notion of cause” (49:1). Syme was not a social scientist but considered that an understanding of both the social and biological was paramount in understanding health and disease and encouraged his students to learn to ask the right questions. Asking the right questions is, however, tricky without genuine interdisciplinary dialogue, parity of perspective, and a shared vision of the social. Too often social science disciplines, such as anthropology, are assumed as under-laborers of medicine, public health, and the life sciences: their methodologies and insights positioned at the bottom of the hierarchy of evidence and constrained by the burden and expectation of epidemiological styles of reasoning and proof (13, 62). This makes finding a common ground difficult.

To embrace the social or indeed the biosocial (92, 93), we need to understand its liveliness—that it is dynamic, relational, and situated in and mediated by politics. Messy as this might appear, we ignore it at our peril. By placing CKDnt in an ecosocial context and embracing its politics, we have an opportunity to understand emergent conditions on multiple scales: we can, for example, examine CKDnt as a disease of global connections—an outcome of modernization, industrial development, neoliberal aspirations of growth, labor and welfare arrangements, capital circulations, and flows of waste and contamination, and so on. In “determinants” speak, these are the “causes of the causes” (56, 94). They intensify the vulnerabilities of the poor and multiply their exposure to harm. However, without large-scale political and systemic change, it is difficult to target intervention so far “upstream”. We might instead focus on local infrastructural arrangements, the kinds of concerns and problems populations encounter on a daily basis and which directly condition health and sickness. These might be considered to operate midstream (61)—as direct, material, encounters with things that do not function well (73). Infrastructural concerns are the traditional terrain of public health (45). They show us that the conditions of disease emergence are locatable, studiable, and can be mitigated. Here anthropologists have a direct role to play not only in ethnographically mapping these conditions, but also in fostering relations with local populations in a spirit of coproduction and shared understanding. How do we make this work? Where to begin?

### Thinking “intervention, otherwise”

We argue that the only place to begin is in local settings and with local populations. This is, however, challenging. Seeberg and Meinert remind us that, despite recognition of the significance of political and structural forces in the rise of chronic conditions worldwide, chronic health remains insistently cached out in terms of behavior and lifestyle^2^. They note:

“[t]he WHO suggests that a ‘major reduction in the burden of NCDs will come from population-wide interventions’, but equally acknowledge that these ‘are not implemented on a wide scale because of inadequate political commitment, insufficient engagement of non-health sectors, lack of resources, vested interests of critical constituencies, and limited engagement of key stakeholders’ (WHO 2011, vii). In the absence of a concerted attempt to fully implement a structural and political approach to intervention, the WHO maintains its focus on healthy lifestyle as the single most important preventive strategy” (82:55).

Perhaps relying on political commitment is part of the problem. Since 2000, Poncitlán communities have attempted to raise awareness and engage relevant authorities with little success. In the course of two decades of witnessing the devasting impact of CKDnt on their communities, no avenues for collaboration between communities and local authorities have emerged. Of course, it goes without saying that, they have been doing this largely in isolation and without the necessary support of the research community. Nevertheless, it is with and from local populations that we find the commitment to live “otherwise”: to find ways of mitigating environmental harms and establish informal modes of care and caregiving.

CKDnt necessitates collaborative modes of authorship and intervention in order to respond to the challenges of diseases with uncertain etiologies and no definitive routes to care. It cannot be left to experts alone. It is critical to engage populations and communities as equal partners in public health interventions rather than tokens in expert-led strategies. This recalls the words of the previously quoted nephrologist interlocutor who remarked that we will not be able to dialyze or transplant our way out of CKDnt. This is not only because we are reaching limits in the supply of biomedical interventions, but also because these interventions are, too, becoming part of the wider problem. Nephrology, is, for example, a major contributor to medical waste, at a level that is disproportionately high compared with many other medical therapies. This is largely owing to the multiple liters of source water used to generate the pure water that becomes dialysate and, which then, for peritoneal dialysis, is packaged in plastic (95). The global dialysis population is projected to reach five million by 2025, and with the corresponding environmental damage only set to intensify, the quest for alternative ways of researching, intervening, and caring is imperative. Community engagement lies at the heart of this. To date, there have been few environmental interventions to mitigate CKDnt. Increased understandings of environmental change and harm can help communities to better cope with the nature of everyday risk and provide relevant authorities with appropriate social and cultural pathways to target interventions.

In summary, a social determinants perspective, in which determinants are conceived as static, reductive, discrete, and ahistorical, provides few clues to the complexities of environmentally induced diseases like CKDnt. This tends to shut down lines of inquiry in advance. By mapping out the social conditions of disease emergence and centralizing vernacular understandings *in situ*, we emphasize the critical importance of place-based, temporally inflected accounts in understanding contemporary environment–health relations. It is by following local interlocutors that we are shown not only where to look, but what to examine and indeed, what to measure, where and how to intervene, as well as with whom. Cause and care are not two sides of health problems but are fundamentally entangled—the conditions of one are the conditions of the other and must be understood in tandem. Collaborative working requires a paradigm shift and a decentering of expertise in order to genuinely co-produce new ways to understand and act on biosocial phenomena. In turn, this requires us to broaden our engagement and collaboration with CKDnt-affected communities living and working in contaminated environments, and work to facilitate and enhance dialogue between affected communities, advocacy and support organizations, research communities, and relevant public authorities with a stake in health and environmental welfare, and also to identify genuinely collaborative, interdisciplinary pathways to knowledge-building and intervention.

## Data availability statement

The original contributions presented in the study are included in the article/supplementary material. Further inquiries can be directed to the corresponding author.

## Ethics statement

The studies involving human participants were reviewed and approved by the Hospital Civil de Guadalajara, Fray Antonio Alcalde (Mexico) and the University of Liverpool. Written informed consent from the participants was not required to participate in this study in accordance with the national legislation and the institutional requirements.

## Author contributions

CK has contributed project conception, data gathering analysis, and writing to this paper. CP-A has been involved in much of the data gathering and analysis that this paper is based on. He has contributed to the writing and editing of the revised draft. All authors contributed to the article and approved the submitted version.

## Footnotes

1 See anthropologists Seeberg and Meinert (82) for a challenge to the distinction between infectious and communicable diseases (CDs) and chronic or non-communicable diseases (NCDs).

2 Chronic conditions have been identified by the WHO as constituting 60% of deaths globally today, with 80% of these occurring in low- and middle-income countries (LMICs) (82).
